# The content and processes of patient-derived quality of care indicators for people living with multiple long-term conditions (MLTC): A scoping review

**DOI:** 10.1177/26335565261451686

**Published:** 2026-05-14

**Authors:** Sara Tavares, Thomas Beaney, Arad Reisberg, Ania Henley, David Belsey, Angela Edwards, Laura E. Downey

**Affiliations:** 1 The George Institute for Global Health, 4615 Imperial College London, London, England; 2 Patient Experience Research Centre (PERC), 4615 Imperial College London, London, England; 3 211065 The George Institute for Global Health, University of New South Wales, Sydney, Australia

**Keywords:** multimorbidity, multiple long-term conditions (MLTC), quality indicators, quality of care, patient involvement, scoping review

## Abstract

**Background:**

The growing prevalence of multiple long-term conditions (MLTC) poses a public health challenge. Existing quality of care (QoC) indicators are poorly suited to the needs of MLTC populations with limited clarity on how quality should be measured. This scoping review aimed to map QoC indicators for MLTC in primary care developed with patient and caregiver input, and to characterise the methods and extent of that involvement.

**Methods:**

Scoping review following the six-stage framework by Arksey and O’Malley refined by Levac et al. Searches were conducted on six databases. Studies were included if adults with two or more chronic conditions were involved. Data were charted on indicator content (name, quality domain, data sources, measurement characteristics) and development processes (methodological approaches and stakeholders involved). Where indicators were not specified, qualitative findings were synthesised to identify QoC domains and mapped to the Donabedian model and Institute of Medicine quality domains. Community partners with lived experience of MLTC were involved.

**Results:**

Twenty-five studies were included, 78 QoC indicators were identified and a further 33 quality domains were synthesised through thematic analysis. Quality was predominately measured through patient-experience surveys rather than indicators. Studies articulated quality through care processes such as care coordination, shared decision-making and holistic assessments. Outcomes focused on functional capacity, social participation and quality of life.

**Conclusion:**

Despite robust evidence on what matters to people living with MLTC, few patient-derived QoC indicators have been developed into measurable indicators. Further work is needed to co-produce indicators suitable to existing primary care settings.

## Background

The epidemic of chronic conditions is a major public health challenge that is projected to continue to rise.^1,2^ Chronic conditions are typically defined as long-term health problems of long duration that can span from years to decades, requiring ongoing management with medications or other therapies and are not usually cured.^3,4^ In western countries, approximately 40-60% of adults have at least one chronic condition, and around half live with two or more long-term chronic conditions, referred to as multimorbidity or multiple long-term conditions (MLTC).^5^ MLTC is commonly defined as the presence of two or more long-term conditions, including physical or mental health conditions, learning disabilities, symptom complexes, sensory impairments, or substance misuse.^6^ These coexisting conditions not only negatively impact quality of life for patients and their caregivers,^7^ but also create complex needs compounded by the interplay of medical conditions, contextual factors and social determinants of health,^8^ often resulting in greater healthcare requirements and utilisation.^9^ Substantial uncertainty exists on what interventions are most effective at improving care for people with multiple long-term conditions (MLTC).^10–12^ Part of this failure is attributable to a global healthcare system structured around single-disease pathways of care,^11–13^ which may have limited applicability to those with MLTC, leading to fragmented care and risk of harmful interactions.^14^

Healthcare quality indicators are measurable metrics of care designed to provide comparative data applicable to health system monitoring, management and policymaking^15^ and are often divided into structures, outcomes and processes of care^16^ categories. However, most indicators are specific to incentivise management of individual conditions, which might not align with the broader needs of patients.^11^ To encourage a shift towards patient-centred care models, in the United Kingdom, the National Institute for Health and Care Excellence (NICE) has produced four quality statements for the care of those with MLTC, emphasising identification of multimorbidity, shared goal-setting, care coordination and medication review.^6^ Furthermore, the use of patient-reported outcomes measures (PROMs) and experiences measures (PREMs) have been increasingly advocated in clinical practice and quality improvement.^17^ However, many PROMs and PREMs were originally developed for research purposes,^18^ primarily to measure health and functional status,^19^ often with limited patient involvement in their design.^11^ Consequently, the constructs they capture may not fully reflect what matters to patients, particularly in the context of MLTC. Variation in their design and psychometric properties further limits their suitability as consistent quality metrics across settings.^20^ Actively involving patients and carers from the outset in the development of quality measures can help ensure alignment with real-world priorities and improve their practical value.^21,22^

This scoping review aimed to map published evidence relating to patient and caregiver-informed quality of care indicators for people living with MLTC. The review examined how quality of care is defined from patient and caregiver perspectives, the content of any existing patient-derived MLTC QoC indicators, and the processes used to develop them, with the aim of clarifying the scope and gaps of the current evidence base to inform future work in developing a set of indicators to be used in primary care.

## Methods

This scoping review followed the enhanced adapted six-step scoping methodological framework by Levac et al.^23^ based on Arksey and O’Malley^24^ with a previously published protocol.^25^ Quality assessment of individual studies was not undertaken, consistent with scoping review methodology, as the aim was to map and synthesise evidence on processes and content of QoC indicators for MLTC, rather than to evaluate methodological rigour.

### Identifying the relevant studies – The search strategy

Search strategies were developed with an experienced librarian and structured using a population, concept and context (PCC) framework. Searches were conducted across six databases: CINAHL (EBSCO), EMBASE (Ovid), MEDLINE (Ovid), PsycINFO (Ovid), Global Health (Ovid), HMIC (Health Management Information Consortium) (Ovid). Filters were applied for human studies, adult populations, English language and publication date from 2000 onwards. Full search strategies are reported in the published protocol elsewhere.^25^ The reference lists of all included studies were screened to identify additional publications of relevance to the research questions. All references were imported into bibliographic manager Covidence^26^ for screening of titles, abstracts, full texts and data extraction. Duplicate citations were removed automatically by the software, with any mismatched duplicates removed manually.

### Study selection

The full study eligibility criteria have been described in the protocol^25^ and summarised in Table 1. The title and abstract of each citation were screened for eligibility by ST and LD using Covidence.^26^ ST and LD conducted independent and blinded full-text review of all studies potentially identified for inclusion, with disagreements resolved through discussion.

**Table 1.** Summarised review inclusion criteria.

Criterion	Description
Population	Adults (18 years old or over) with diagnosed or self-reported MLTC, as defined by the National Institute for Health and Care Excellence (NICE) as the presence of two or more long-term conditions, including physical or mental health conditions, learning disabilities, symptom complexes, sensory impairments, or alcohol or substance misuse.^6^
Concept	Studies identifying, developing or validating QoC indicators involving patients and/or caregivers. If not developing indicators, we considered studies eliciting patients’ and/or caregivers’ priorities within the context of MLTC care or specific components of MLTC care (i.e. coordination of care).
Context	No country-related exclusion, but only studies published in English that reported on primary care or outpatient settings were included.

### Data collection and charting the data

All data extraction was performed using Covidence by one author (ST), and to ensure consistency between researchers, 10% of included studies were randomly checked for accuracy by the second author (TB). Our data charting approach included study characteristics and extraction of qualitative findings, along with illustrative examples**.** Where available, data on quality indicator content were extracted, including indicator name, quality domain, data sources, measurement characteristics, and stakeholders involved in their development.^27^ Additional data were extracted on participant characteristics, study design and level of patient and caregiver involvement, mapped against Carman et al.'s framework, which outlines a continuum of patient and public involvement across direct care, organisational design and policy making contexts.^28^ Fortnightly research meetings between authors (ST and LD) were held to discuss data extraction, resolve discrepancies, and ensure consistency in the interpretation of findings.

### Collating, summarising, and reporting the results

**Figure 1.**
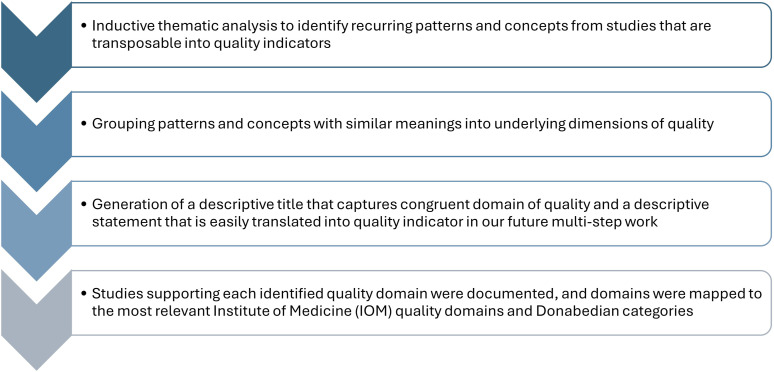
Qualitative data analysis and transformation.

Studies were analysed for conceptual similarity and overlap by one author (ST).^29,30^ Studies that explicitly defined or developed QoC indicators or patient-experience survey metrics, were grouped and reported in a summarised in a tabular format (S1 – Table 4). Where studies did not develop formal QoC indicators but instead elicited participants’ perspectives on care quality or priorities, an inductive thematic analysis was conducted to generate the main domains of quality of care (see example of codebook on S1 - Table 5). Qualitative data from included studies were synthesised into operational definitions of quality aspects in MLTC care to inform future indicator development work (Figure 1).^31^ This distinction is maintained throughout the results section to avoid conflating conceptual domains of quality with validated indicators. As the aim of this review was to identify processes and content of QoC indicators, attributing quantifiable metrics (denominator and nominator) when these were not reported by primary studies was not appropriate.

To ensure robustness and credibility, the inductive themes were verified by all authors through extensive discussions with our community advisory partners. To facilitate their relevance and applicability in broader healthcare contexts, the domains of quality identified were presented for their matching categories of the Donabedian framework on quality of care^16^ and the six domains of healthcare quality by the Institute of Medicine (IOM): safe, effective, patient-centred, timely, efficient, and equitable.^32^ Where domains of quality could be mapped to different IOM domains, only the most relevant IOM domain judged by the authors was assigned. Although the Donabedian framework does not incorporate specific patient factors, it is one of the most well-known and used frameworks in quality improvement efforts.^33^ It categorises quality into structure (the healthcare system and organisational context in which care is delivered), process (the delivery of care and interactions between patients and providers), and outcomes (the effects of care on patient health and experience).^16^

### Patient and public involvement

Five members of the North-West London community with lived experience of MLTC who had registered to contribute to research through the Imperial College London Patient Experience Research Centre (PERC) were part of the core research team. These members guided the study’s conception, scope, evidence review, synthesis, and write-up. A summary of their contributions to thematic analysis and discussion is provided in S1- table 7.

## Results

### Study selection

**Figure 2.**
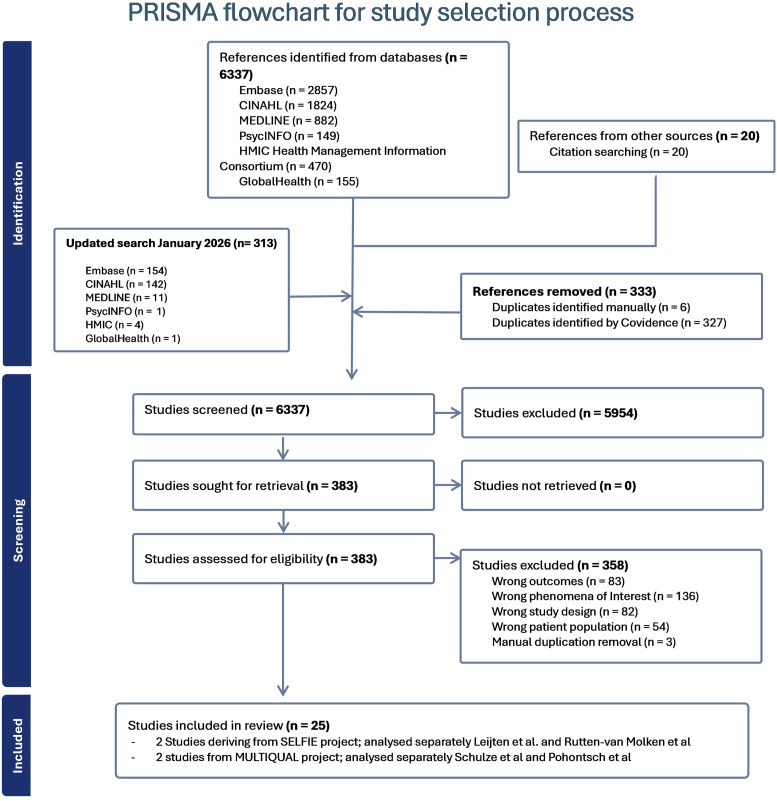
PRISMA flowchart.

Database searches were first conducted in September 2025 and updated in January 2026 (see Figure 2). The initial search yielded a total of 6337 studies, and the update identified a further 313 records. 20 articles were identified for full-text screening through second-level screening. 383 full-text studies were assessed for eligibility and 25 met our inclusion criteria. 358 were excluded either by focusing on the outcomes of specific interventions designed for MLTC, on single chronic condition indicators, validation of instruments or emphasising methodological rigour rather than capturing patients or caregivers’ insights on quality of care. The characteristics of the full-text articles included in the study as presented in detail on S1 file - Table 1. Included studies were published between 2008 and 2025 and conducted across a range of countries and health system contexts, including the United Kingdom, Australia, Canada, South Africa, United States of America, South Africa, Thailand and several European countries.

### Participants’ characteristics and level of involvement

All studies included patients and/or caregivers with varying degrees of patient involvement. A total of 7201 patients, 1625 caregivers, and 2609 stakeholders were included in the selected studies. All studies included participants with both physical and mental health conditions. Patient ages, when reported, ranged from 18 to 94 years old with most between 65 and 84 years. Some caregivers were also patients, and when their relationship to patients was reported, they were mostly spouses or adult children. Participant characteristics are detailed in S1 file - Table 2. Reporting varied substantially across studies, limiting comparability and quantitative synthesis.

Most studies employed qualitative methodologies, including interviews and focus groups, to elicit patient and caregiver perspectives on what constitutes good-quality care in the context of MLTC. A smaller number of studies adopted more iterative designs that blended multiple participatory methods such as individual or group interviews alongside expert panels to engage patients as true collaborators in producing the findings.^34–37^ Levels of patient and caregiver engagement varied across studies, ranging from consultation to partnership and shared leadership. S1 file – Table 3 maps the studies methodology approach to the patient engagement framework by Carman et al.^28^

Healthcare professional stakeholders were either participants in the research themselves^35,38–41^ or recruited to be part of a panel of experts.^35,37,42–45^ These comprised healthcare professionals from multiple disciplines, professional roles and areas of practice, health system leaders, policymakers, researchers, health economists, health insurance payers, and information technology experts.

### Patient-derived QoC indicators in MLTC

A total of 78 mostly disease-agnostic, patient-centred QoC indicators or metrics relating to MLTC care were extracted from the literature and are summarised on S1 – Table 4. These were collated from studies that developed formal quality indicators,^34,35,46^ quality improvement metrics from guidelines,^45^ or structured experience or satisfaction tools.^44,47–49^ While data were lacking for some indicator items, without a comprehensive description of their meaning or attributing a measurable numerator and denominator,^46^ most indicators and metrics demonstrated relevance and face validity within their original studies. This was largely because they were grounded either in prior systematic reviews of the evidence with extensive input from patients and/or professional experts during consensus and prioritisation stages.^35,44–48^ While most indicators were grounded in qualitative data from patients and caregivers, some were still supported by expert input only, such as “identification of patients with multimorbidity” and “monitoring adherence to treatment” indicators.^35^

Two studies identified the need and relevance to continue having specific single-disease metrics as part of quality assessment for MLTC.^45,46^ One of these, focused on specific single-disease quality indicators, prioritising cardiovascular-related guidelines and outcomes of care, such as blood pressure, diabetes, cholesterol management. However, when subject to a qualitative discussion with patients, more person-centred and non-disease specific measures emerged such as emotional well-being, physical function as an outcome of care and personalised approached to medication reviews.^45^ Overall, there was a balance between the number of patient-related indicators, such as communication, shared decision making and holistic reviews, and organisational indicators. For the latter, timeliness of care, waiting times and accessibility to care were commonly present either as formal indicator of care,^46^ or as part of quality experience surveys.^44,48,49^

Although descriptions of indicators are provided, feasibility on the operationalisation of these indicators in existing clinical records and practice continues to be heterogeneously reported, highlighting the challenge in measuring nuanced person-centred care delivery.^35,46^ For most of the indicators captured, a large proportion were suggested to be captured via patient-reported experience surveys.^35,44,47–49^

### Quality domains represented across patient and caregiver perspectives

For the primary studies using qualitative approaches to elicit priorities and conceptual meaning of quality, findings were thematically aggregated as quality-of-care domains, reflecting aspects of care considered meaningful rather than fully operationalised measures. Complementary qualitative illustrative quotes for each domain are provided in S1 - Table 6.

Across all included studies, 33 quality domains were identified and mapped into organisational structures, clinical processes, and care outcomes.^16^ Although our synthesis shows that people affected by MLTC place greater weight on the processes of care such as respectful, holistic, coordinated, and consistent interactions based on continuity, there is still value on traditional outcome or disease-based metrics. Independence in daily living and maintaining quality of life emerged as core outcome priorities, outweighing classical endpoints such as mortality or longevity. After analysis of domains relating to individual or specific-disease physical symptoms, our co-authoring community partners suggested a broader “physical outcomes” domain, instead of individual symptoms such as pain, breathlessness, fatigue. This allows recognition of the wide range of possible symptom experiences in MLTC. A similar approach was applied to mental health domains, avoiding condition-specific labels (e.g. depression) to reflect the spectrum of psychological experiences reported by participants.

**Figure 3.**
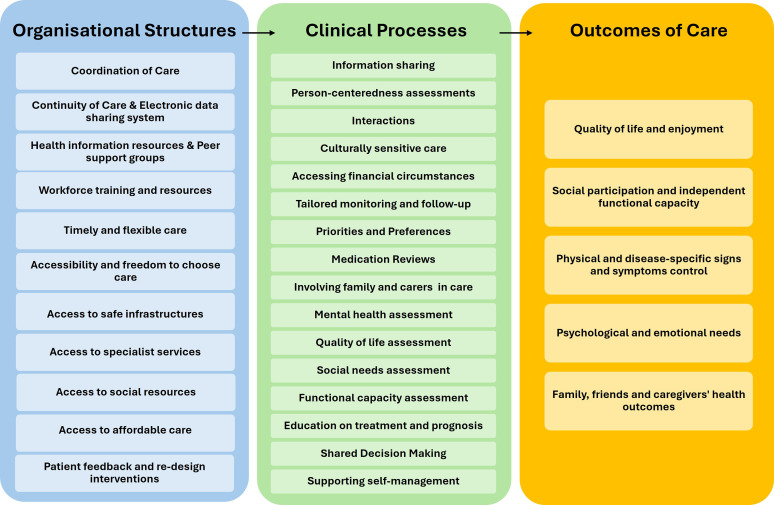
Summary of QoC domains grouped by organisational structures, clinical processes and outcomes of care.

The community partners also suggested that although the indicator “identification of patients with multimorbidity” was not directly reflected in qualitative accounts, it was closely aligned to having an electronic or shared medical record that facilitates care coordination and continuity. Having a responsible professional or service, where all health-related data are kept, such as General Practitioner records, facilitates this identification. A summary of thematic analysis discussions with community partners is provided in S1 - Table 7. Figure 3 provides a summarised overview of the quality domains identified.

Both extracted indicators and quality-related concepts consistently identified bio-psycho-social elements of care, such as coordination, continuity, shared decision-making, medication reviews, holistic assessment, and tailored follow-up. Considerable heterogeneity in terminology and operational focus was observed, reflecting the use of different conceptual models and frameworks to define and analyse quality of care across studies. For example, Schulze et al.,^35^ Giusti et al.,^38^ Rijken et al.,^50^ Bayliss et al.,^46^ and Fradgley et al.,^47^ develop study-specific conceptual frameworks of chronic care management or quality based on qualitative findings. In contrast, other studies^42,44,48,50^ explicitly adopted existing frameworks of chronic care management or established models of high-quality care.

The largest number of quality domains matched the Donabedian category of clinical processes (n=17), followed by organisational structure (n=11) and outcomes of care (n=5). Domains relating to care outcomes, could also be delineated as clinical processes. For instance, assessing quality of life during a consultation represents a clinical process. A similar distinction applies to health-related behaviours. Promoting healthy lifestyles and providing patient education are processes of care, while subsequent knowledge acquisition, self-management capability, and behavioural change represent outcomes. Discussions with our co-authoring community partners emphasized that such distinction is crucial when operationalising quality in MLTC.

Among the IOM quality domains, indicators were more commonly matched with Patient Centred (n= 14), Effective (n =7), Equitable (n=5), Timely (n= 1), Safe (n=6). Table 2 displays indicator titles and descriptions, mapped to the relevant IOM quality domains and Donabedian categories relating to organisational structures. Tables 3 and 4 summarise domains relating to clinical processes and outcomes of care, respectively.

**Table 2.** Domains of quality related to organisational structure (physical, organisational and human resources).

Quality of Care Domain Title	Studies	IOM	Content/Description
Coordination of Care	18^34–36,38,40–42,46,47,50–57^	Patient-Centred	Having access to a responsible healthcare professional or informal caregiver^34,35,38,40,54,55,57^ with clear responsibilities, tasks and roles^36,38,41^ capable of coordinating all information sharing between healthcare professionals. This professional is a a point of contact^40^ to effectively manage health appointments and advocate for patients.^53^ Examples of this coordination includes coordinating community, social support resources,^47^ providing support in times of crisis^54^ (physical and mental health) and after discharge from hospital.^36,37^
Continuity of Care supported by an electronic data sharing system	11^36–38,41,42,46,50,52,56–58^	Patient-Centred	Being seen by a familiar healthcare provider over time,^37,38,41,52,56–58^ someone who knew their history and needs well, avoiding to have to repeat their health history.^38,56–58^ When seeing different healthcare professionals, continuity of care could still be achieved by having a widely available electronic health record system where all data is shared and accessible between different healthcare professionals involved in patient’s care.^36,38,42,50,56^
Health information resources and peer support groups	4^38,42,49,56^	Patient-Centred	Signposting to a list of trustworthy sources^49^ or having access to a patient portal, e-health tools or assistive technologies^42^ in a clear, free-of-jargon manner.^56^ Having access or be able to participate in patient and family support groups was also seen as informative and supportive to navigate health needs.
Workforce training and resources	12^34–38,40–42,54–56,58^	Effective	Training programmes to healthcare staff, social care professionals and informal caregivers was highlighted to advance the quality of care for chronic and complex conditions.^35,41,42^ Examples of training included triage, geriatric care, polypharmacy management,^41^ conducting care plans,^41^ psychology and communication skills,^34,40^ motivational interviewing,^37^ care coordination,^37^ duty of care and confidentiality.^56^ Specifically for nursing home settings, training needs were identified for nurses to assess medical conditions, communicate and raise relevant questions to GPs.^41^ To allow care to deal with MLTC complexity, participants also recognised the need to have a fair allocation and division of responsibilities, allocated time and the human resources to provide good care in primary care.^36,38,54,55,58^
Timely and flexible care	10^36,38,41,42,44,46,49,54,56,57^	Timely	Services are available promptly when required,^44,56^ particularly in times of health crisis and based on clinical urgency,^38^ reducing waiting times to see healthcare professionals or being informed on estimated wait.^36,49^ To facilitate this, the included participants in the studies highlighted the need for administrative processes and systems to allow convenient appointment scheduling,^49,57^ and flexible re-scheduling due to their unpredictable disease trajectory.^54^
Accessibility and freedom to choose care	12^34,36,38,41,42,46,49,52,54,56–58^	Equitable	Having access to nearby healthcare facilities,^52,56^ car parking facilities nearby,^49^ or access to home visits for those who lack access to transport due to poor mobility, difficulty in travelling.^34,38,52,54^ Availability of care also meant having the freedom to choose healthcare providers and geographical locations.^36^ Care access, particularly near to home, appeared to be more important to frailer and elderly patients with functional limitations when compared to younger and those with more years of education. The latter group valued personal familiarity and continuity of care by a provider that knew them well over convenience.^38,41,52,56–58^
Access to safe infrastructures	4^36,38,44,56^	Safe	Safe facilities in relation to nearby crime levels, communicable diseases and infection control measures,^38^ clean and comfortable facilities,^36,44^ and up-to-date equipment particularly in rural to semi-rural areas.^56^
Access to specialist services	4^34,53,56,57^	Effective	Having access to specialist services when needed, recognising the need for specialist expertise at time, but preferably in an integrated manner with one person coordinating all information and writing referral letters.^34,53^ Some studies suggested a “one stop” clinic, where all healthcare professionals were located in one place to reduce travelling burden.^56,57^
Access to social resources	7^37,38,40,42,46,54,58^	Equitable	Offer, draw attention or refer to social and community-based services and resources^37,38,42,54^ that are meaningful to patients and are able to fulfil more than one role ideally, such as improving quality of life, health or social needs.^58^
Access to affordable care	5^36,38,43,46,56^	Equitable	Services that are accessible by everyone regardless of socioeconomic backgrounds.^38,43^ This includes affordable medications, consultations, mental health crisis services^56^ and health insurance for countries where this is applicable.
Patient feedback and re-design interventions	3^37,40,47^	Patient-Centred	Increase opportunities and initiatives for patients, caregivers to give feedback and include patient’s ideas and concerns in service re-design projects.^37,40,47^

**Table 3.** Domains of quality related to clinical processes (methods and procedures).

Quality of Care Domain Code	Studies	IOM	Content/Description
Information sharing between specialities supporting consistent advice	8^34,38,40,41,47,55–57^	Safe	Information sharing between primary care and specialities^34,38,40,47,55^ allows continuity and coordination of care^47,56,57^ and this should be compounded in respectful communication between professionals^38,41^ who provide consistent advice and information to patient’s needs. Writing a treatment plan on a collective digital patient record used between healthcare professionals facilitates this.
Person-centeredness assessments	13^34,36,37,44–47,50,53–55,58^	Patient-Centred	Having an holistic approach on medical and psychosocial aspects of health,^34,36,45^ allows discussion of situation as a whole^,50,58^ according to patient’s needs, prior history and preferences^47^ not focusing only on clinical guidelines^58^ physical symptoms or single-diseases.^44,54,55^
Interactions between patients, caregivers and professionals	10^36,38,40,44,46,52–54,57,58^	Patient-Centred	Being able to discuss concerns and fears,^58^ ask questions freely,^38^ receive information that is free-of-jargon^40,54^ without being rushed, taking time to verify understanding and allowing people to feel as equals and listened to.^38,54,57^ Professionals actively listen,^36^ are approachable and reliable,^58^ friendly,^54^ caring,^57^ helpful, respecting and polite.^44,52,53^
Culturally sensitive care	5^38,45,46,54,56^	Equitable	Communication and care is planned respecting individual’s financial status, ethnicity, cultural background,^45,54^ gender and^38^ religion.^56^
Assessment of financial circumstances	5^35,36,38,46,52^	Equitable	Assessing financial needs and support requirements^35^ during consultations is particularly important for those with fewer resources, those who rely on insurance^52^ or are unable to work.^36,38^
Tailored monitoring and follow-up	9^34,37–39,42,49,50,56,57^	Patient-Centred	Different perspectives on how disease progression should be monitored were identified among participants across the included studies.^34,38^ Frequency of follow-up requires tailoring to patients’ needs, condition and preferences^38,39^ and can adopt many approaches, such as after test results,^49^ as an early intervention and prevention,^42^ after a hospital discharge,^56^ frequent monitoring of disease progression,^34,38^ whenever the GP thinks it’s necessary^50^ or when patients feel they need it beyond their self-management capabilities.^50,57^
Priorities and Preferences in Care	10^35,38,40,41,45–47,53,55,57^	Patient-Centred	Discussing and documenting on medical records patient’s priorities, goals and preferences.^40,47^ This includes general goals in life, overall meaning of life^41^ and what it means to live with disease,^38,53^ repercussions for aging and future,^45^ and overall complex diffuse issues.^55^
Medication Reviews	12^34–36,40–42,44–46,51,55,56^	Safe	Medication reviews should actively involve patients by providing information about medications prescribed,^35,41^ side-effects and how to manage them.^51^ It should be tailored to patient’s needs,^34^ involving caregivers whenever appropriate and explaining potential drug-interactions, reviewing indications and exploring any new treatments available.^56^ Assessing positive desired effects from medications versus unwanted side-effects or medication reactions is fundamental for patients.^35^ The burden of taking too many different medications for different conditions should be considered by weighing the potential benefits against side-effects that could lead to more health problems.^40,45,51^ Medication management and reviews go beyond prescribing the correct medication for the diagnosis.^36,42,44^ It needs to consider individualised treatment goals,^45^ balancing between short-term effects and long-term consequences,^45^ assessing the need for medication (dis)continuation.^55^
Family, friends, caregivers’ involvement in care	7^34,35,38,42,52,54,56^	Effective	With patient's agreement, informal caregivers should be involved in the process of care decision-making options,^35,42^ providing them with information about diagnosis, symptoms, side effects.^38^ Caregivers concerns^34^ and their involvement in the different approaches on how to manage safety need to be acknowledged.^54^
Mental health assessment and screening	2^35,36^	Safe	Mental health and depression targetted reviews, need to be given equal importance to physical symptom management during clinical contacts, even when not the main focus of the follow-up review.^36^
Quality of life assessment	6^35,36,38,39,45,53^	Effective	Documenting quality of life from a functional, psychological and social perspective,^35,38,39,45^ often described by participants as the ability to engage with their lives and realistic activities.^36,53^
Social needs assessment	4^38,42,58^	Safe	Assessing the level and type of social support network patients have access to, considering this can come from family, friends or neighbours.^38,42,58^
Functional capacity assessment	8^38,42,43,45,51,53,54,58^	Effective	Assessing ability to carry out normal roles, tasks and future life goals.^38^ This includes the ability to engage with social activities, live independently,^53^ do a hobby^42,45,51^ Assessment of safety might require balancing interventions and strategies between maintaining safety and overly restrictive practices.^54^
Providing proactive treatment and prognosis education	2^49,56^	Safe	Proactive sharing of information about treatment options available and different services available^56^ how to handle medical emergencies and contact details on who to contact first.^49^ Participants across the included studies had varying notions on how comprehensive information about disease progression should be provided. Whilst some wanted to be fully informed and in-charge of their own care,^52^ others highlighted that negative information on disease or poor prognosis should not be routinely provided, unless prompted by the patient.^38^
Shared Decision Making with patients	13^34–38,40,41,45,50–52,54,57^	Patient-Centred	Making a collaborative decision with patients beyond being informed, seen as a key process to support autonomy.^37^ During this process, patients can be given the freedom to make final decisions, regardless of decision complexity, on treatment burden, medications,^50^ treatment options and plans,^54,57^ specialist referrals, diagnostic and procedure tests.^36,51^ Patients’ responsibility for the diseases and care plans must be discussed based on participant’s lived experiences,^38,57^ individual needs,^45^ values and preferences,^34,36^ and their own background.^45,52^ Discussions include potential harms, risks, consequences,^38^ benefits,^35^ and trade-offs of treatment options.^40^ The extent to which patients valued being involved in the shared decision-making process varied across the participants in the included studies, depending not only on individual characteristics such as background demographic, health literacy, medical and functional characteristics^52^ but also the cultural context where they lived.^38^
Shared Decision-making involving carers	2^38,41^	Patient-Centred	Involving family, friends and informal caregivers when patients agree to this. For nursing home residents, they expressed the need to be able to discuss problems directly with GPs without interference from a relative or others.^41^
Supporting self-management and patient education	10^34,35,37,39,42,49–51,53,57^	Patient-Centred	Being informed about their conditions and provided information on coping, self-management strategies towards health and care,^34,35,39,42,50,51^ symptoms and long-term effects of diseases and medications^49^ whilst checking provision and evaluation of knowledge attained.^34,50^ Self-management includes managing psycho-emotional and physical symptoms^53^ in opportunistic times such as discharge from services^37^

**Table 4.** Domains of quality related to outcomes of care (end results of healthcare services on patient health and well-being).

Quality of Care Domain Code	Studies	IOM	Content/Description
Quality of life and enjoyment	6^35,36,38,39,45,53^	Patient-Centred	Quality of life from a functional, psychological and social perspective,^35,38,39,45^ often described by participants as the ability to engage with their lives and realistic activities.^36,53^ A specific important characteristic of quality of life, was the ability to continue enjoying life, defined as having pleasure and happiness in life.
Social participation and independent functional capacity	8^36,38,42,43,45,51,53,58^	Patient-Centred	Participants in the included studies found it important to continue to be socially engaged in society despite their disease.^36,58^ For this, participants highlighted the need to be able to carry normal daily living that allow one to engage in social activities, hobbies^38,42,45,51^ and live independently.^53,58^ Although both patients and caregivers expressed preference on outcomes focusing on physical function and energy,^45^ in frailer individuals, the balance between achieving independence and restricting actions that prioritise safety and prevention of accidents (e.g., falls) was not always aligned between patients and caregivers.^54^
Physical and disease-specific signs and symptoms control	7^35,38,39,45,46,51,52^	Effective	Health-related symptoms and their burden^35,52^ on a person’s ability to live according to their priorities and goals was a key aspect of care.^38^ A systematic assessment of disease or symptom-specific aspects of care may not be appropriate for all individuals.^46^ Accounts from included study participants described the impact of symptoms on daily functioning, including chronic and neuropathic pain,^35,39,45^ fatigue and energy levels, poor sleep, weight control,^39^ gait imbalance, shortness of breath, dizziness.^51^
Psychological and emotional needs	6^36,43,45,46,52,58^	Effective	Assessing psychological, emotional and mental health needs for those with MLTC,^43,45,58^ especially during new diagnosis and adjustment period,^36^ offering support to those who want it.^52^
Family, friends and caregivers’ health outcomes	4^36,38,54,56^	Patient-Centred	Assessing the impact of caregiver’s responsibilities over their overall health, (ability to work, emotional and psychological wellbeing, financial burden) and providing support according to their needs. The type and level of involvement of caregivers in care, although importantly highlighted, appeared to vary according to patients’ own ability to self-care and degree of involvement in their own care.^52^

## Discussion

This scoping review mapped and characterised published evidence relating to patient and caregiver informed quality of care indicators and quality domains for people living with multiple long-term conditions (MLTC) in primary care and outpatient settings. While a substantial body of literature explored what quality of care means from patient and caregiver perspectives, a more limited number of studies explicitly developed or operationalised patient-derived QoC indicators for MLTC, highlighting the important need for the development of such indicators to align clinical practice with individual experience and priorities.

A key finding of this review is the marked imbalance between the richness of conceptual work describing patient and caregiver priorities and the scarcity of formally developed, patient-derived QoC indicators ready for implementation. Other reviews examining quality indicators in other healthcare settings and medical specialities^17,31,59^ have also found challenges in the identification of existing quality indicators that are consistently defined in identifiable, measurable and validated.

For the included studies, one multi-step programme developed MLTC-specific indicators, with well-defined units of measurement (numerators and denominators),^35^ according to the current accepted methods of quality indicator development.^27,60,61^ With the exception of another study by Bayliss et al., that explored the possibility of using electronic medical records for indicator measurement,^46^ quality was often inferred through survey instruments designed to measure patient-reported experiences.^44,47–49^ To develop these indicators, authors adopted a multi-step approach using expert panels and focus groups with patients, caregivers and stakeholders^34,35^ and subsequently tested them for validity, discriminative capacity and feasibility.^62^ Although approaches to QoC indicator development vary, there is increasing recognition that robust indicators should demonstrate at the very least importance, appropriateness, and validity. Multi-step approaches integrating patient and caregiver perspectives with expert panel consensus methods provide a strong methodological foundation for indicator development.^27,60^

Notably, when disease-specific metrics were subjected to qualitative exploration with patients,^45^ more person-centred and non-disease-specific priorities emerged, such as emotional well-being, physical function, treatment burden, and personalised medication review. As one participant reported they *“… found it important that healthcare providers look at patients as a whole (…) People like it when a healthcare provider pays attention to them, in addition to following protocols*”.^58^ This divergence highlights a persistent tension between what is currently measurable within existing health system infrastructures and what matters most to people living with MLTC. Others have also highlighted an excessive reliance on processes and outcome measures for single conditions,^63,64^ suggesting that quality of care can appear higher when using disease-specific indicators, when compared to more patient-centred outcomes.^65^

Overall, the captured quality domains identified in this review align with existing quality statements for MLTC care, which include personalised care planning based, coordination of care, and regular medication reviews.^6^ From a measurement perspective and indicator development efforts, this means that processes of care are largely measured through structured data contained in electronic health records, whilst organisational quality domains are measurable through policies and health organisation documents.^16,61,66^ Despite agreement in overall quality domains, differences in how health and healthcare quality are perceived amongst individuals were prominent and are a reflection of individual’s characteristics, across time and the social contexts where they reside.^67,68^ For example, while shared decision-making was widely valued, preferences regarding the degree and timing of involvement varied substantially. Some participants in the included studies wished to lead decision-making conversations, whereas others preferred to delegate responsibility to clinicians or family members.^52^ This heterogeneity should be interpreted as the need to produce flexible measurements on MLTC care quality, giving patients the freedom to set their own care priorities. These findings underscore the challenge of developing generic and non-disease specific indicators that are sufficiently flexible to accommodate diversity without becoming so broad as to lose operational meaning.

Across both extracted indicators and synthesised quality domains, clinical processes of care were more frequently emphasised than organisational structures or outcomes of care. Domains relating to shared decision-making, medication review, holistic assessment, and tailored follow-up were consistently identified across studies, despite substantial heterogeneity in conceptual framing. Although participants have highlighted the importance of organisational domains of quality, such as workforce training and resources to support coordination and continuity of care, these were framed as measures to improve clinical processes. This emphasis on processes of care aligns with the realities of MLTC care, where quality is often experienced through ongoing regular interactions, rather than through discrete clinical endpoints.^69^

When discussing outcomes of care, patients and caregivers in the included studies consistently prioritised maintaining independence, functional capacity, social participation, and quality of life (QoL) over traditional biomedical outcomes such as disease progression or mortality. From a quality of care indicator perspective this creates another practical challenge. Although QoL is often considered a key outcome for patients, it is typically harder for services to influence it when compared to processes of care delivery.^60,70^ Evidence suggests that the relationship between care processes, patient-reported experience and QoL is inconsistent and varies across clinical settings. In primary care, associations between patient-reported experience and outcomes tend to be weak,^71^ whereas integrated outpatient models have reported improvements in QoL.^72^ This may be a reflection of how care is delivered in these settings. Primary care involves repeated contacts in which outcomes are shaped by multiple clinical and social influences, whereas outpatient care is often more targeted at specific conditions or symptoms. Evidence from settings with more frequent interactions with healthcare staff, such as nursing homes, further suggest that interpersonal processes of care seem to have a stronger relationship with QoL outcomes than organisational-related domains of care.^73^ QoC measurement in MLTC should therefore account for both patient characteristics and care settings. Our community partner co-authors further emphasised that assessing patient-related outcomes such as QoL or mental health should be recognised both as a process of care through regular and meaningful assessment and as an outcome reflecting the impact of interventions at different points of time. Even when patient outcomes are the focus for improvement efforts, process and organisational indicators remain important, particularly where they can be meaningfully linked to outcomes that matter to patients.

In this context, patient-reported outcome measures (PROMs) and patient-reported experience measures (PREMs) offer an important bridge between traditional QoC metrics and patient-centred outcomes and experiences.^74^ Although the application of PROMs and PREMs in theory is appealing, and has gained increased importance in both research and policy contexts, their application as metrics of care quality remains limited.^19,75,76^ Generic QoL measures such as the widely used EuroQoL EQ-5D may pose problems for people with specific needs who may be less able to complete them or whose perspectives are not adequately captured in existing instruments.^20^ Their use may therefore risk widening health inequalities, by excluding under-represented groups. For those living with MLTC, PROMs may lack sensitivity to detect change when used to evaluate complex, coordinated, and multidisciplinary interventions.^77^ Indeed, interventions designed to improve holistic and integrated care seem to enhance patients’ perceptions of care quality but show inconsistent effects on health-related QoL when assessed through standard PROMs.^78–80^ More broadly, the use of PROMs or PREMs as quality metrics presents significant implementation challenges, as data collection often places additional burden on patients without being routinely embedded within existing electronic health record systems.^81^

In sum, for people living with MLTC, QoC measurement requires a systems-thinking perspective, whereby quality should be conceptualised not as a sum of isolated processes indicators, but as an emergent property of complex systems where outcomes depend on relationships and contextual factors, moving away from fragmented metrics detached from patient priorities.^82^

## Strengths and limitations

Our scoping review used transparent methods previously reported in a published protocol.^25^ A key strength in our review was the explicit focus on patient and caregiver perspectives and its evident distinction between operational indicators and conceptual quality domains. The involvement of community partners throughout the synthesis strengthened the interpretive validity of findings and ensured alignment with lived experience. Limitations include reliance on published studies in English and variability in reporting across included studies, which limited direct comparison and data pooling. As a scoping review, methodological quality of studies was not assessed, and findings should be interpreted as mapping and synthesis rather than endorsement of specific measures.

## Conclusions and implications for future indicator development

This scoping review mapped published patient and caregiver-informed QoC indicators and quality domains for people living with MLTC in the context of primary care. Across the literature, quality was often articulated through processes of care such as coordination, continuity, shared decision-making and holistic assessment and typically measured using patient-experience surveys. Relatively few studies translated patient and caregiver priorities into well-defined, operational and ready-to-use QoC indicators suitable for primary care.

The limited availability of well-defined, patient-derived QoC indicators for MLTC reflects a research to practice gap rather than a lack of understanding of what matters to patients and caregivers. Given the heterogeneity of MLTC experiences, future indicator development should move away from single, fixed indicator sets and instead support flexible approaches that allow indicators to be selected or tailored according to individual patient priorities and care contexts.

Limited attention has been given to how QoC indicators for MLTC can be implemented and embedded into routine practice, particularly in relation to data availability, workforce capacity, and integration within existing healthcare systems. The methods identified in this review, including qualitative elicitation of patient priorities usually followed by structured consensus approaches, provide a foundation for developing indicators with stronger validity, grounded in both professional expertise and patient experience. Our future work will focus on validating key quality domains through qualitative interviews, followed by a multi-round Delphi consensus with patients, clinicians, and system leaders. We will refine, prioritise and operationalise measurable indicators that can be integrated within routine care pathways, leveraging existing electronic health record systems.

## Supplemental material

Supplemental Material - The content and processes of patient-derived quality of care indicators for people living with multiple long-term conditions (MLTC): A scoping review Supplemental Material for The content and processes of patient-derived quality of care indicators for people living with multiple long-term conditions (MLTC): A scoping review by Sara Tavares, Thomas Beaney, Arad Reisberg, Ania Henley, David Belsey, Angela Edwards, Laura E Downey in Journal of Multimorbidity and Comorbidity

## Data Availability

All relevant data are within the paper and its supporting Information files.*
