# Supplementary material for: The content and processes of patient-derived quality of care indicators for people living with multiple long-term conditions (MLTC): A scoping review
Source: J Multimorb Comorb. 2026 May 14;16:26335565261451686. doi: 10.1177/26335565261451686 (PMC13180098; doi:10.1177/26335565261451686)
Supplement: Supplemental Material - The content and processes of patient-derived quality of care indicators for people living with multiple long-term conditions (MLTC): A scoping review [file sj-pdf-1-cob-10.1177_26335565261451686.pdf]

Supplementary Information

Table 1. Study Characteristics 2
Table 2. Patient (P), caregivers (C) and stakeholders (S) characteristics 8
Table 3. Engagement of patients and caregivers and study designs across selected studies according to Carman et al framework 13
Table 4. List of Quality-of-Care Indicators identified 14
Table 5. Example of inductive coding analysis 18
Table 6. Complementary illustrative quotes for identified domains of quality 21
Table 7. Discussion of review findings with community partners 26
References for this Supplementary file 28

Table 1. Study Characteristics

Authors	Year	Country	Setting	Aim	Recruitment strategies	Data Analysis
Simpson et al.(1)	2023	United Kingdom (UK)	Primary Care (community dwelling)	To elicit and prioritise determinants of improved care in people with multiple conditions.	Purposive and snowball sampling, via online forums, social media and websites of local government adult social care and voluntary sector groups	Qualitative deductive analysis mapped to SELFIE theoretical framework and inductive analysis for Items outside of framework
Giusti et al.(2)	2022	Jordan, South Africa and Thailand	Hospital based clinics, primary care (GP clinics) and specialist non-governmental centre	To appraise and adapt a person-centred care (PCC) model across three serious physical conditions to generate an evidence-based framework and recommendations	Purposive sampling of participants known to a hospital-based clinic or inpatient	Deductive thematic analysis mapped to on Santana et al PCC framework and authors' systematic review findings. Inductive analysis for concepts outside the framework. Derived components of PCC presented as structures, processes and outcomes (Donabedian model)
Schulze et al. *[1] (3)	2022	Germany	Primary Care (GP clinics)	To develop a set of generic quality indicators for the management of patients aged 65 and older with multimorbidity	Random sampling of patients selected via participating GP surgeries. Patient representatives recruited from patient organizations	NGT consensus process by online rating of candidate QoC indicators, analysing the proportion of agree or strongly agree votes (> 75% in all categories) followed by open voting discussion
Pohontsch et al.*(4)	2021	Germany	Primary Care (community GP clinics)	To identify relevant aspects of quality of care to patients and match them to a literature-based set of QIs.	Participants selected via GPs; snowballing sampling of patients asked to invite family members	Qualitative content analysis. Deductive codes from author's previous systematic review and expert panel. Inductive coding for new aspects of quality.
Tinetti et al.(5)	2021	United States of America (USA)	Primary care (GP clinics)	To describe the outcome goals and health care preferences of this population with MLTCs using facilitated sessions documented into electronic	Screening of healthcare records of patients registered in a GP practice	Descriptive statistics for patient characteristics. Outcome goals and health care preferences obtained from personalised care templates on electronic health records, aggregated into the

*[1] different publications of the same overall multi-step project (MULTIQUAL)

				health records		predetermined categories
Rijken et al.(6)	2021	Netherlands	Primary Care (community dwelling)	To describe the perspectives and priorities of multimorbid patients with care preferences	Qualitative: Random sampling from a national database of chronic illness and disability.	Qual component: Thematic deductive analysis based on the Integrated Multimorbidity Care Model. Inductive analysis for new data emerged.
					Survey: Purposive sampling of patients with chronic conditions from 14 General Practices	Survey: Univariate statistics for demographic characteristics and to assess preferences for care.
Sasseville et al.(7)	2021	Canada	Primary Care (GP clinics)	To describe patients and healthcare professionals' perspectives on a patient-centred care intervention	Participants selected via GP	Deductive thematic analysis from the author's previous scoping review
Kuipers et al.(8)	2020	Netherlands	Primary Care (healthcare insurers clinics)	To identify the views of patients with multimorbidity on what is important from patient centred care	Purposive sampling from participants known to intervention GP practice	Combined quantitative (factor arrays) and thematic analysis of interviews based on factor arrays decisions.
Rimmelzwaan et al.(9)	2020	Netherlands	Primary Care (GP clinics)	To explore patients with multimorbidity perspectives on general practice; To assess their care experiences and the impact of chronic conditions on daily functioning	Purposive sampling from participants known to 3 GP practices	Thematic analysis using constant comparative methods
Pereira et al.(10)	2020	Australia	Primary Care (care coordination service by a regional public health service)	To explore patients' experiences living with chronic and complex needs of care coordination	Purposive sampling from care coordination service known to authors	Descriptive thematic analysis

Rutten-vanMolken et al. **[2] (11)	2020	Austria, Croatia, Germany, Hungary, Netherlands, Norway, Spain, UK	Primary Care (GP and hospital outpatient clinics, nursing home residents, community dwelling)	To measure patients' preferences of integrated care outcomes in multimorbidity and to compare preferences between patients, partners and other stakeholders.	Patients and caregivers: purposive and convenience sampling from internet networks and support groups, clinic waiting rooms, patients known to researchers. Healthcare professionals': convenience and snowballing sampling. Stakeholders: convenience, purposive and snowballing sampling from own networks, healthcare insurers, social care municipalities departments	Statistical analysis: Differences in the mean preferences of stakeholder groups evaluated using scale heterogeneity multinomial logit (S-MNL) models
Leijten et al.**(12)	2018	Austria, Croatia, Germany, Hungary, Netherlands, Norway, Spain, UK	Primary care (community dwelling)	To understand good health and well-being; and what is the most important (top 10 list) in good care processes of care for people with multimorbidity.	Convenience sampling and snowballing via authors networks and patient organisations.	Focus groups notes analysed via thematic analysis, using an initial inductive approach followed by a deductive approach according to Triple Aim structure (population health, patients experience with care and costs)
Heslop et al.(13)	2019	Australia	Primary Care (care coordination service by a Local Healthcare Network)	To gather understanding on patients and HCPs experiences with care coordination, identifying areas for improvement	Purposive sampling, patients, caregivers and HCPs known to care coordination service	Thematic analysis with the EBCD steering group. Creation of a video with selected touch points covering contextual information, client footage illustrating touch points and staff quotes to support consumer footage. Video then used in a joint EBCD event, where clinicians and consumers identified areas for service improvement and prioritisation
Kuluski et al.(14)	2019	Canada and New Zealand	Primary Care (GP clinics)	To capture and outline attributes and characteristics of good care	Convenience sampling through HCPs working at selected sites	Direct content analysis approach with in-depth inductive and line-by-line analysis of codes,

**[2] different publications of the same overall multi-step project (SELFIE)

				and what matters to older people with complex care needs and their caregivers		organising similar text into core categories.
Manga et al.(15)	2018	South Africa	Primary Care (GP clinics)	To develop and validate a patient-reported experience measure (PREM) tool to assess self-reported patient experience of chronic care.	Purposive sampling	Qual: inductive thematic analysis.<br>Survey: Cronbach's alpha to determine internal consistency (reliability)
Ferris et al.(16)	2018	USA	Primary Care (community dwelling)	To elicit perspectives of patients, caregivers and stakeholders on modifiable factors leading to inappropriate care for older adults with multimorbidity	Purposive sampling, participants from a previous multistakeholder group known to the authors	Constant comparative method
Bennett et al.(17)	2017	USA	Primary care (community dwelling)	To identify two high-priority clinical questions for those with multimorbidity and understand patients and family caregivers perceptions of meaningful outcomes to inform harm assessments	Purposive sampling, participants from the Kaiser Permanente National guideline programme	Thematic analysis
Fradgley et al.(18)	2016	Australia	Hospital outpatient clinics	To identify patients' preferences among a set of change initiatives and actions for patient-centred quality improvement	Convenience sampling of participants in outpatient clinic waiting rooms	Descriptive statistics on demographic characteristics. Analysis of participants allocation of points across 2–5 proposed quality initiatives, Average allocation of points across 2-5 quality initiative were analysed and totals were compared to identify relative priorities
Schiotz et al.(19)	2016	Denmark	Hospital outpatient clinics	To identify opportunities for improving care by understanding how patients experience care coordination	Purposive sampling, through screening of medical records	Manifest qualitative content analysis
van de Pol et al.(20)	2015	Netherlands	Primary Care (Nursing home, community	To explore the views and needs of healthcare professionals and older patients	Purposive sampling for nursing home residents and community	Constant comparative analysis and thematic analysis

			dwelling)	and identify areas for improving on primary care	dwelling. Convenience sampling for nurses and GPs known to residents.	
Sav et al.(21)	2015	Australia	Primary Care (GP clinics, community dwelling)	To investigate the healthcare priorities of patients with chronic conditions and their carers. To evaluate differences between them, and if priorities differ depending on geographical location	Purposive sampling and snowballing sampling, via community pharmacies, general practices, health agencies, government and non-government organisations	Quantitative list of individually ranked healthcare priorities, followed by thematic analysis by grouping similar priorities. Calculation of votes given to themes for final prioritization.
Singer et al.(22)	2012	USA	Primary Care (GP clinics)	Develop and pilot a new survey instrument to measure the integration of patient care as experienced by patients.	Random sampling from electronic medical records	Fisher's exact test assessed differences between full sample and respondent sub-group. Logistic regression model of unit nonresponse using fixed effects for covariates. Covariance matrix of variables to identify survey's latent structure. Cronbach's Alpha for internal consistency measurement for each identified factor
Bayliss et al.(23)	2008	USA	Primary care, not-for-profit Health Maintenance Organization	Explore processes of care desired by elderly patients with multimorbidity, informing the development of future care improvement interventions	Random sampling from a 357 sample of a respondents to a previous survey	Constant comparison method
Bayliss et al(24)	2014	USA	Primary care, not-for-profit Health Maintenance Organization	To inform the development of a data-driven measure of quality care for individuals with multiple chronic conditions derived from an electronic health record (EHR).	Purposive sample of patient participants. No description of sampling strategies for experts participating.	Inductive thematic analysis of webinar transcripts. Descriptive analysis of prioritisation survey for each possible quality metric presented
Tušek-Bunc et al (25)	2025	Slovenia	Primary Care (GP clinics)	To assess the quality of primary care delivered to patients with CCs in Slovenia and to identify factors associated with perceived care quality	Nested sampling framework: All Slovenian practices invited to participate. Patients randomly invited to participate.	Quantitative multilevel analysis of patient-reported quality of care using validated survey

Table 2. Patient (P), caregivers (C) and stakeholders (S) characteristics

Authors	P sample (n)	P Age (mean, range)	P female gender (n, %)	P ethnicity, education (n, %)	Conditions (diagnosis)	C Sample (n)	C characteristics (age, relationship)	C female gender (n, %)	S sample (n)	S characteristics
Simpson et al.(1)	2	ns*	ns	ns	2 or more chronic conditions (ns)	ns (not specified)	ns	ns	22	Health and social care professionals, data scientists, researchers
Giusti et al.(2)	86	Range: 22-81<br>Mean: 54.75	39, 45.3%	50 Jordan, 22 South Africa, 14 Thailand<br><br>Secondary school: 18, 21%	Advanced cancer, Chronic Obstructive Pulmonary Disease (COPD), Heart Failure (HF)	49	Range: 19-69<br>Relationship: ns	39, 79.6%	54	21 doctors, 28 nurses, 2 pharmacists, 1 pharmacy manager, 1 operations manager, 1 clinical manager
Schulze et al.* (3)	47	ns	ns	ns	2 or more chronic conditions (ns)	9	Relationship: Spouses and adult children	ns	23	General practitioner, geriatrics, nurses, social workers, physical therapists, and pharmacists. Health economists, researchers and patient representatives
Tinetti et al.(5)	163	Mean: 77.6	109, 66.9%	158, 94% white<br><br>College: 59, 36.2%	Arthritis, Atrial Fibrillation (AF), COPD, Depression, Diabetes, HF, Hypertension (HTN)	none	none	none	none	none
Pohontsch et al.*[1](4)	29	Range: 65-84	11, 37.9%	ns	2 or more chronic conditions (ns)	9	Range: 49-78<br><br>Relationship: Spouses and adult children	3, 33.3%	none	none

Rijken et al.(26)	Qual 20	Range 40-89<br>Mean: 68.2	13, 65%	ns	Diabetes Mellitus Type 2 (T2DM), Ischemic Heart Disease (IHD), Cancer, Arthritis, Asthma, COPD, Multiple Sclerosis, Thyroid, Anxiety Disorder, ADHD, Depression	none	none	none	none	None
	Survey 863	Range: 22-96<br>Mean: 70.5	440, 50.9%							
Sasseville et al.(7)	9	Mean: 55	4, 44.4%	College: 6, 66.7%	2 or more chronic conditions (ns)	none	none	none	18	9 nurses, 2 General Practitioners, 4 nutritionists, 2 physical therapists, 1 respiratory therapist.
Kuipers et al.(8)	16	Range: 56-88<br>Mean: 72	7, 44%	College: 13, 81%	Asthma, Diabetes, COPD, Heart, Vascular Disease	none	none	none	none	None
Rimmelzwaan et al.(9)	12	Range: 47-87, Mean: 72.7	7, 58 3%	ns	Hypertension, T2DM, Peripheral Artery Diseases, AF, Hypothyroidism, Stroke, COPD, Psoriasis, Asthma, Breast, Colon, Bladder and Prostate Cancer, Chronic Musculoskeletal and pain conditions, Squamous Cell Carcinoma, Basal-Cell Carcinoma	none	none	none	none	none
Pereira et al.(10)	6	Range: early 60s – late 70s	3, 50%	ns	COPD, HF, Musculoskeletal conditions, chronic pain	none	none	none	none	none
Rutten-van Molken et al.** (11)	1314	Mean: 54	667, 50.7%	Low education: 373, 28.3%<br>Medium education: 526, 40%	Depression, anxiety, chronic back pain, T2DM, gastric conditions, cardiovascular disease	1425	Mean age: 47.4<br>Relationship: ns	769, 53.9%	2383	1210 not specified HCP<br>1173 stakeholders from health-insurance companies, departments paying for

										social care, politicians, public servants, ministries of social care or local governmental bodies
Leitjen et al.**(12)	58	Range: 37-86<br>Mean: 65.3	28, 48.3%	ns	Cardiovascular, metabolic, gastric, respiratory, skin and subcutaneous tissue, eye, ears and mastoid diseases, Nervous system diseases	none	none	none	none	none
Heslop et al.(13)	14	Range: 59-95	ns	ns	COPD, T2DM, Parkinson's disease, Alzheimer's, dementia, orthopaedic and cardiac conditions, post-polio	ns	ns	ns	13	Registered Nurses, Occupational Therapists, Physiotherapists, Pharmacists, Social Workers, Drug, Alcohol clinicians.
Kuluski et al.(14)	83	Range: <50 to > 75	59, 71%	Non-English speakers: 8, 10%<br>Maori: 24, 29%<br>East Asian: 14, 17%	COPD, Cancer, Asthma, HTN, IHD, T2DM, High Cholesterol, Stroke, Arthritis, Dementia, Alzheimer's, Chronic Pain, Anxiety, Depression	89	Range age: <50 to > 75<br>Relationship: adult children, spouse	none	none	none
Manga et al.(15)	Qual 29	Range: > 18	ns		HTN, T2DM, Asthma, HIV, Osteoarthritis, Rheumatoid arthritis, IHD	none	none	None	7	General medicine doctor, Family physicians, from a primary, district and tertiary public health setting
	Survey 200	Range: 23-82	152,							
Ferris et al.(16)	6***	ns	ns	ns	2 or more chronic conditions (ns)	6***	ns	ns	31	Primary care physicians, advanced practice nurses, specialty clinicians, health system leaders and payers, healthcare design and information technology experts, representatives of

										national organisations
Bennett et al.(17)	27***	ns	ns	ns	Coronary artery disease, HTN, HF, high cholesterol, T2DM, stroke, transient ischemic attack (TIA), peripheral arterial disease (PAD), dementia	27***	ns	ns	ns	Leaders from a national integrated cardiovascular guideline program
Fradgley et al.(18)	541	Mean 60.2	279, 51.6%	Aboriginal islander: 21, 3.9%<br><br>Secondary school or less: 270, 49.9%	2 or more chronic conditions (ns)	none	none	none	none	none
Schiotz et al.(19)	14	Mean: 71.3 Range: 49-88	10, 71.4%	ns	COPD, heart disease, T2DM, depression, nephropathy, glaucoma, Parkinson's disease, cancer, back disease	none	none	none	none	None
van de Pol et al.(20)	53	Mean: 83.36 Range: 70-94	35, 66%	ns	2 or more chronic conditions (ns)	none	none	none	41	Nursing home nurses, General Practitioners
Sav et al.(21)	43 (including 20***	Mean: 57.6	15, 65.2%	Non-English speakers: 6, 26%<br><br>Aboriginal islander: 8, 34.8%	2 or more chronic conditions (ns)	42 (including 20***[3]	ns	15, 68%	none	none
Singer et al.(22)	527	Range: 18-over 75	347, 65.8%	Non-English speakers: 80, 15.2%	2 or more chronic conditions (ns)	none	none	none	none	none

***[3] patients were also caregivers

				White: 314, 59.6%<br>Black: 77, 14.6%<br><br>Secondary School: 148, 28%						
Bayliss et al.(23)	26	Range 65-84	13, 50%	Secondary school: 8, 30.8%	Between 4-16 chronic conditions with minimum diabetes, depression and osteoarthritis.	5	ns	ns	none	none
Bayliss et al.(24)	10	Range 70–87	Ns	Ns	With 3 or more of 10 common chronic conditions: hypertension, congestive heart failure, hyperlipidemia, diabetes mellitus, coronary artery disease, chronic obstructive pulmonary disease, osteoarthritis, osteoporosis, depression, obesity.	none	none	None	17	Experts in clinical geriatrics and primary care, health policy, quality assessment, health technology, and health system operations
Tušek-Bunc et al (25)	3003	Range 55-74	1883, 62.7%	Higher education: 38.3%	Ns specific conditions;<br>49.3% had one chronic condition<br>28.3% had two<br>22.4% three or more	Ns	ns	ns	ns	81 participating GP practice providers, participants not individually described

Table 3. Engagement of patients and caregivers and study designs across selected studies according to Carman et al framework

Patient engagement level based on Carman *et al* framework(27)	Methodology and study reference
**Consultation**<br>(participants provide input but decisions remain clinician-driven)	• Qualitative Interviews (5, 7, 8, 10, 14, 16, 19, 23)<br>• Focus groups (20)<br>• Mixed-methods combining interviews with surveys(9) or focus groups(6)
**Involvement**<br>(participants actively collaborate in decision-making, patients as advisers, or their recommendations used to inform funding decisions)	• Six-staged experience based co-design (13)<br>• Qualitative interviews (2)<br>• Expert Panel only (21)<br>• Discrete choice experiments (11)<br>• Patient surveys based on prior qualitative work to define conceptual framework for survey elements (18, 22)
**Partnership and shared leadership**<br>(participants are equal partners in shaping policies, research, and care delivery)	• Using different methods of data triangulation, through multi-step approaches:<br>○ expert panel + interviews + questionnaires (15);<br>○ systematic review + qualitative work + prioritisation techniques (3, 4, 12) with subsequent validation / survey testing (25);<br>○ Focus groups + interactive webinars + Delphi process involving patients (24);<br>○ Delphi-process followed by patient focused groups (17)

Table 4. List of Quality-of-Care Indicators identified

Indicator Name, (reference)	Numerator and Denominator	Indicator Domain	Stakeholders	Data Source[4]
Proactive Pain Assessment, (3)	Y	Patient Factors	Experts, patients and caregivers	Y
Screening for depression, (3)	Y	Patient Factors	Experts, patients and caregivers	Y
Identification of patients with multimorbidity, (3)	Y	Patient Factors	Expert Panel	Y
Involving partner, family and caregivers, (3)	Y	Patient Factors	Experts, patients and caregivers	Y
Addressing financial support needs, (3)	Y	Patient Factors	Experts, patients and caregivers	Y
Monitoring adherence to treatment, (3)	Y	Patient Factors	Expert Panel	Y
Facilitating patient education and self-management, (3)	Y	Patient Factors	Experts, patients and caregivers	Y
Quality of life assessment, (3)	Y	Patient Factors	Experts, patients and caregivers	Y
Assessment of symptoms burden	Y	Patient Factors	Experts, patients and caregivers	Y
Assessment of biopsychological support needs¸(3)	Y	Patient Factors	Experts, patients and caregivers	Y
Eliciting patient's preferences, (3)	Y	Patient Factors	Experts, patients and caregivers	Y
Patient-Provider communication, (3)	Y	Patient Factors	Experts, patients and caregivers	Y
Mutual agreement on treatment's goals, (3)	Y	Patient-provider communication	Experts, patients and caregivers	Y
Information about potential benefits and harms of treatment options, (3)	Y	Patient-provider communication	Experts, patients and caregivers	Y
Information about medication, (3)	Y	Patient-provider communication	Experts, patients and caregivers	Y
Shared decision making, (3)	Y	Patient-provider communication	Experts, patients and caregivers	Y
Assessment of treatment burden, (3)	Y	Patient-provider communication	Experts, patients and caregivers	Y
Medication Review, (3)	Y	Patient-provider communication	Experts, patients and caregivers	Y
Monitoring of pain management, (3)	Y	Patient-provider communication	Experts, patients and caregivers	Y
Written treatment plan, (3)	Y	Patient-provider communication	Experts, patients and caregivers	Y
Documentation of adverse drug reactions, (3)	Y	Patient-provider communication	Experts, patients and caregivers	Y
Regular updates of medication plan, (3)	Y	Patient-provider communication	Experts, patients and caregivers	Y
Assigning responsibility for coordination of care, (3)	Y	Context and organizational structures	Experts, patients and caregivers	Y
Comprehensive care documentation, (3)	Y	Context and organizational structures	Experts, patients and caregivers	Y
Training programs addressing the management of patient with multimorbidity,	Y	Context and organizational structures	Experts, patients and caregivers	Y

[4] Data source: information is given on how to measure the indicator (e.g. electronic records, patient survey, interviews with patients)

(3)				
Assess caregiver support at home(24)	N	Deliver contextually relevant and compassionate care	Expert Panel	Y
Shared decision-making, (24)	N	Deliver contextually relevant and compassionate care	Experts, patients and caregivers	Y
Advance care planning, (24)	N	Deliver contextually relevant and compassionate care	Expert Panel	N
Address social determinants, (24)	N	Deliver contextually relevant and compassionate care	Expert Panel	N
Understand patients' perceptions of themselves with MCC¸(24)	N	Deliver contextually relevant and compassionate care	Experts, patients and caregivers	N
Care guided by patient goals and priorities, (24)	N	Respect patients	Experts, patients and caregivers	Y
Address patient questions, (24)	N	Respect patients	Patients	Y
Consider patient finances, (24)	N	Respect patients	Patients	Y
Patient experience of care, (24)	N	Respect patients	Experts, patients and caregivers	Y
Care Coordination, (24)	N	Avoid Harm	Experts, patients and caregivers	N
Readmissions, (24)	Y	Avoid Harm	Experts, patients and caregivers	Y
Functional Assessment, (24)	N	Avoid Harm	Experts, patients and caregivers	N
Medication reconciliation, (24)	N	Avoid Harm	Expert Panel	N
Use of potentially inappropriate medications or doses, (24)	N	Avoid Harm	Expert Panel	Y
Selected disease-specific metrics, (24)	N	Provide high-quality condition-specific care	Expert Panel	Y
Depression assessment, (24)	N	Provide high-quality condition-specific care	Experts, patients and caregivers	Y
Anticipatory management of clinical needs (laboratory studies and annual flu / pneumonia vaccine), (24)	N	Provide high-quality condition-specific care	Patients	Y
Pain assessment, (24)	N	Provide high-quality condition-specific care	Expert Panel	Y
BMI assessment, (24)	N	Provide high-quality condition-specific care	Expert Panel	Y
Timeliness of care, (24)	N	Minimize logistical barriers to care	Patients	Y
Access to care, (24)	N	Minimize logistical barriers to care	Patients	N
Informational continuity between providers, (24)	N	Optimize patient–clinician communication	Patients	Y
Customizing communication	N	Optimize patient–clinician	Patients	Y

to patient preference, (24)		communication		
Interpersonal continuity of Care, (24)	N	Optimize patient–clinician communication	Experts, patients and caregivers	Y
Effective team-based care, (24)	N	Optimize patient–clinician communication	Expert Panel	N
Accuracy of EHR documentation, (24)	N	Optimize patient–clinician communication	Patients	Y
Wasted resources, (24)	Y	Optimize efficiency	Experts, patients and caregivers	N
Cost of Care, (24)	N	Optimize efficiency	Patients	N
Billing inefficiencies, (24)	N	Optimize efficiency	Patients	Y
Patient Perceptions of Integrated Care Survey Experience (22)	N	Coordination of care within, across and between teams	Patients	Y
	N	Continuity: familiarity with patient over time	Patients	Y
	N	Continuity: proactive and responsive action between visits,	Patients	Y
	N	Patient-centredness	Patients	Y
	N	Shared responsibility	Patients	Y
Patient-reported experience measure (PREM) tool (15)	N	Patient Experience Satisfaction	Patients and experts	Y
	N	Respectful Care	Patients and experts	Y
	N	Staff taking interest in patient's health	Patients and experts	Y
	N	Listening to patient's problems and worries	Patients and experts	Y
	N	Patient's priorities	Patients and experts	Y
	N	Promoting patient role on own health	Patients and experts	Y
	N	Medication support	Patients and experts	Y
	N	Continuity of Care – familiarity with the same professional	Patients and experts	Y
	N	Waiting times	Patients and experts	Y
Blood Pressure management, choosing treatments and targeting levels (17)	N	Prevention of adverse long-term health outcomes	Patients and experts	Y
Diabetes management, choosing treatments and sugar levels (17)	N	Prevention of adverse long-term health outcomes	Patients and experts	Y
Cholesterol management, choosing tailored treatments and cholesterol levels / goals (17)	N	Prevention of adverse long-term health outcomes	Patients and experts	Y
Coronary artery disease, blood thinners and medications (17)	N	Prevention of adverse long-term health outcomes	Patients and experts	Y
Physical function and energy as an outcome	N	Not specified	Patients and experts	N

of care (17)				
Emotional health and well being (17)	N	Not specified	Patients and experts	N
Interaction with providers and health care system (17)	N	Not specified	Patients and experts	N
Avoidance of treatment burden, risks and side effects (17)	N	Not specified	Patients and experts	N
40-item Primary Care Practice Questionnaire (PCPQ) (25)	N	Organizational characteristics: location, service availability, staffing, care coordination, planning, goal setting, and self-management support	Patients and experts	Y
Primary Care Service User Questionnaire (PCUQ) (25)	N	Patient-Reported experience measure question, health status, socio-demographic characteristics, and lifestyle behaviours.	Patients and experts	Y
Quality improvement initiatives questionnaire, (18)	N	Access to care and services: Parking, ease to contact clinic, convenient appointment times, reduce waiting times	Patients and experts	Y
	N	Information: Up-to-date with treatment and condition progress, information at home, manage medical emergencies	Patients and experts	Y

Table 5. Example of inductive coding analysis

Quality Domain Label	Description on primary studies by authors, supported with participants quotes	Study
**Continuity of Care**	Preference in seeing the same HCP over time, avoiding patients to have to go through their health history. But some said they could see different HCP as long as patient information was stored accessibly for every HCP	Giusti et al
	Continuity of care is seen as important, and if needed they don't mind telling their stories several times. Vulnerable patients dislike re-telling their stories due to communication struggles: *"It would be nice if I didn't have to tell them every time. Every time I come, every time, I have to say; I have these medicines."*	Kuipers et al
	Participants value a primary healthcare professional that knows them to avoid to have to repeat their health history. Continuity allows smooth transitions between caregivers and no waste of time. *"I think my general practitioner knows what I am like. I have been his patient for a long time now. He knows me well and that helps in advising me. It would be frustrating if my general practitioner did not know me so well and I had to explain my situation to him all over again"*<br>Payers and/or policymakers assigned lower values to continuity of care (HU, NL and NO), and resilience (DE, ES, HU and NL) than patients. Of the two experience outcomes, continuity of care was generally valued higher than person-centredness.	Rimmelzwaan et al.
	Being seen by the same professional was vital to good quality care in care homes, but frequent unavailability of coordinating nurses threatened this: '*We keep getting different caregivers. One shows up in the morning to help with the elastic stockings. Then there's another one for the medicines*.'	Van de Pol et al.
	Seeing the same healthcare professional avoided having to repeat one's personal health all the time - *"It comes back to [us] explaining things over again about your personal health and everything else. Consumer"*	Sav et al
	Continuity meant a proactive and responsive action between visits and post-visit Information, focused on continuity with a patient following an office visit.	Singer et al
	Participants view continuity more important over convenience, some chose a less convenient location or wait a longer period of time to see a preferred provider: "*Dr. L moved ... to be closer to where she*	Bayliss et al

	lived... So we decided since Dr. L had been through so much of this stuff and taking care of us ... we would rather stay with her. That's the reason we came down here."<br>Participants preferred access to a provider who knew them well and most needed specialists, with the later posing logistical problems.	
	" (...) doctors (...) can't give in-depth time (...) but team people know what worked for this one and that one (...) because of back up team and the input of knowledge they know from working with people on the ground."	Heslop et al
**Quality Domain Label**	**Description on primary studies by authors, supported with participants quotes**	**Study**
**Person-centeredness assessments**	Includes having a holistic view on health problems and needs. Not only treating physical symptoms but overall medical and psychosocial aspects of care	Pohontsch et al.*
	Participants wanted the opportunity to discuss whole situation and not just only medical conditions - "*everything is connected*". To provide points of action for prevention, either for behavioural responses of people with multimorbidity themselves or for appropriate treatment or care provided by professionals.	Rijken et al.
	Too much focus on clinical guidelines. A complete overview of health missed during specific disease follow-up and participants want HCP to focus on their functional state, limitations in daily life and well-being: "*I found it important that healthcare providers look at patients as a whole. I am having trouble with that protocol thing. People like it when a healthcare provider pays attention to them, in addition to following protocols*"	Rimmelzwaan et al.
	Person-centred and authentic, were expressed as "being listened to", "being heard", "feeling understood" and led to trust, empathy and respect - *"I really needed help to express what I wanted [regarding health treatments at end of life] and um [Care Coordinator] understood it. [They] didn't try to talk me out of what I wanted, umm [they] listened to me and [they] wrote it down as I wanted it"*	Pereira et al
	Being approached as a whole person and treated holistically: *"I wish that people treat me in a respectful manner, because it's true, I am sick, but the disease is not me. I don't want to be reduced to my diseases."*	Leijten et al
	Providers being present (listening intently), asking probing questions beyond illness and physical symptoms	Kuluski et al

	Care that does not focus on one chronic condition only that is beyond just a conversation, It matters that the right diagnosis is made, examination and: "*we like to be touched, we like to be examined, like now it's just now a conversation I would say, I feel sometimes if doctor was attending me and you just look on the chart and you write*"	Manga et al
	Need for providers to understand their unique situations and to match treatments to individual patient needs, prior history, having MCCs, race/ethnicity and personal/home situation.	Bennett et al
	Division of care into specialist areas leads to neglected problems or more general problems being overlooked: "*I think you are very much one condition and one condition. But you are several conditions, right? Sometimes I think that it is a bit irritating that you are not [perceived] as a whole person… No one has been able to put it all together and make it come together. I haven't felt that…*"	Schiotz et al
	Delivering care that is not only coordinated but also patient-centered (patients' needs, preferences, and the important role that patients and family members play as active participants in care.	Singer et al
	˜Treats us as individuals, Understands our concerns", "Someone was there for me"	Heslop et al

Table 6. Complementary illustrative quotes for identified domains of quality

Quality of Care Domain Title (Structures)	Participant Quotes
**Coordination of Care**	*"I think when you determine that the patient has COPD…you need to allocate in a specific uhm… caregiver, mentor…I don't know quite what the word is, in banking it would be a relationship banker…and I don't know that that person needs to be a qualified doctor, in fact probably not. But there needs to be somebody who can liaise with them, the doctors, but at the same time is going to be prepared to listen to you. And in fact, have more time to listen to you"*- **Patient**(2)
**Health information resources and peer support groups**	*"Would be nice if we could help each other. For us too to help. To have something that we do in order to show other people that even us, we can do this, we just need to persevere. Yes, for us to persevere and not lose hope* - **Patient***(2)*
	"Well, that printout that Dr. I does (helps me), because many times you talk about so many different things that you don't remember" - **Patient**(23)
**Workforce training and resources**	*So, I think it would be a very good care model if we had, for example, registered physicians who specialize in geriatrics. I think it would be a good care model if we had general medical training, with additional specialization, for example, in mental health. I would prefer geriatrics and that these doctors with such training, who also have many, many or mostly older patients, are given more time(...)* - **Patient***(4)*
	"GPs nowadays have less time for their patients than back in the days. Recently, I visited my general practitioner and mentioned that I had two physical complaints. The general practitioner told me that there was only time scheduled for one complaint. In the past, this would have never happened. There is not much the current general practitioners can do about it. It is a consequence of increasing time pressure. However, it is difficult for patients too." - **Patient***(9)*
**Accessibility and freedom to choose care**	*"Availability also had different definitions per country; (…) this was treatment, care and provider availability and the freedom to choose between them"* – **Author's description of patient participation** (12)
**Timely and flexible care**	*" I just would like doctors to be more readily available. You often phone a doctor now when you're sick and they say, sorry, we're fully booked and can't get you wait until next week"* - **Carer**(21).
	"*I can never tell how my physical condition is going to be from day to day, so I frequently have to cancel and*

	reschedule at the last minute (...)" – **Patient***(14)*
**Access to safe infrastructures**	*Yes, I have a problem with that (traveling to the GP practice). I can get there, but I have to leave my mobility scooter outside. Then I have to go upstairs with the elevator and then I have to walk a bit. And a bit in the waiting room and to the toilet as well. I can't do that*."- **Patient**(8)
**Access to specialist services**	*"Well as a patient I think it's really important to have a relationship between the primary care physician and the specialty care physician so that they trust each other, respect each other."* – ***Patient(16)***
	"My pulmonologist saved my life. – ***Patient (16)***
**Access to social resources**	*"She definitely helps me, things that I am entitled to that I would never had known about and she's brought them to my attention*"- ***Patient***(14)
**Access to affordable care**	*"The law here states that foreigners are charged more than citizens, so it is not discrimination, it is just the law"* - **Patient**(2)
	"This cycle is probably the last one I am gonna take because I can't afford any more. I can barely get my children bread… - ***Patient** (2)*
**Patient feedback and re-design interventions**	*"There's kind of a balance there, to get too far downstream of designing a solution without having a patient voice informing it…can often result in processes of redesign (that are ineffective)"* - **Patient advocate***(16)*

Quality of Care Domain Title (Processes)	Participant Quotes
**Information sharing between specialities supporting consistent advice**	*"Yes, you have a giant infection or you have a lot of infection'. Then the next day another doctor comes and says, 'Well, this is not something we need to treat*"- **Patient**(19)
**Person-centeredness assessments**	*"I think you are very much one condition and one condition. But you are several conditions, right? Sometimes I think that it is a bit irritating that you are not (perceived) as a whole person… No one has been able to put it all together and make it come together. I haven't felt that…"*- **Patient**(19)
**Interactions between patients, caregivers and professionals**	*"(...)When I go to my doctor, I must indeed feel myself in such a relaxed way that I can and dare say anything. Even if they don't agree, or I don't agree with them, it has to be possible to talk with each other"* - **Patient***(8)*
**Culturally sensitive care**	*"Do you (have different recommendations) for different ethnic groups, like Native Americans, and different races? 'Cause I'm American Indian, and diabetes is really rampant (…)My (cholesterol) prescription didn't*

	match me. So I don't take it (…) It's too high."- **Patient**(17)
**Assessment of financial circumstances**	*"Fear, fear for one day having to give up your work for example: if I don't manage any more, if I am unable to work anymore, then we do not have the economic resources to live here anymore"*- **Patient***(12)*
**Tailored Monitoring and follow-up**	*"If you take these medications regularly and then say: We'll have a general check-up once every two years I think that's not enough. I think it should be every year."*- **Patient***(4)*
**Priorities and Preferences in Care**	*"Well ... not everybody is alike. And not all diseases are alike ... I'm not so sure you could devise an overall plan for people with multiple problems. It depends on the kind of person that they are and what the problems are. But you could work individually with each one and find what works best for them, but not as an overall plan."* - **Patient***(23)*
**Medications Reviews**	*"You can have the best health service in the world, if I don't know about it; it may as well not exist."* - **Patient***(21)*
	"They gave me an anti-inflammatory. But that anti-inflammatory… created some other problems. Subsequently, knee replacement was the answer to quiet the arthritis, but the arthritis…is still in the body and is not going to go away." - **Patient***(17)*
	"To me, taking all the medications that are recommended for all of these chronic illnesses we all have—the side effects outweigh the possibility of recovery. Because the side effects are 16 pages long for this one little thing you are on…so you open the door to a whole bunch of other things you didn't even have."- **Patient***(17)*
**Family, friends, caregivers' involvement in care**	"*Perhaps this could be discussed in a phone call with relatives, so that I am informed about the current situation, what is pending or how the medication maybe, yes, what I have to pay attention to as a caring relative and, as I said, if this is okay for my mother*" - **Carer**(4)
**Mental health assessment and screening**	*"Yeah, for me I'd like the mental health to be bigger… for me all my healthcare professionals see the (physical disease) as the big thing with me, I don't, I see my depression as the big thing because that's what affects me day to day."* - **Patient***(12)*
**Quality of life assessment**	*"(the care coordinator) was helping me in trying to get, to get out of the house more so that I can get involved with people you know, what have like got issues like me. They discussed with me about oh what things I can get involved with and what I can look out for, you know?"* - **Patient***(10)*
**Social needs assessment**	*"In Syria, I would be surrounded by my family and relatives, but here I have no one."*- **Patient**(2)
**Functional capacity assessment**	*"I want to go down to the dining room to eat and socialize each day", I want to continue to babysit my*

	grandchildren every day"- **Patient***(5)*
**Clear care documentation on treatment plans**	*"I think to keep an overview of the attending specialists is actually very important. Because when I go to the eye specialist or ear, nose and throat specialist, and the GP doesn't even know this, and usually doesn't get a medical report from them either, then he is actually missing out on important information*" - **Patient**(4)
**Providing proactive treatment education**	*"I need it in writing, because I tell you what, I have a lousy memory. And when you're talking to me over the phone, I don't usually write all this stuff down*" - **Patient**(23)
**Shared Decision Making with patients**	*"She (the provider) gives me good advice. She keeps me informed, she doesn't insist on anything. I am the one who decides. If I am not feeling good about something, she helps me find something else."* - **Patient** *(14)*
	"But *I just could not function. So we changed. (My doctor) said, ok, we'll work it another way…but I said no more of them, because the lungs was the hardest part, once they found out I had COPD and that was why I was going around sounding like a horse all day long. (…) I got from the beta blocker for treating my large heart (…) just made me feel worse"*- **Patient***(17)*
**Shared Decision-making involving carers**	"*Most of the time they will listen, they will respond, we will make a decision together*" - **Caregiver**(2)
**Supporting self-management and patient education**	*I now have some tricks for doing physical activity without getting a hypoglycaemia*", *"I have a healthcare team, but part of the power is mine (…)."* - **Patient**(7)
**Quality of Care Domain Title (Outcomes)**	**Participant Quotes**
**Quality of life and enjoyment**	*Even if I still have the disease, I want to wake up in the morning, make coffee, I don't have to cook, I can fix myself something simple, move around the house. I want to reduce the pain so I can be around (my family) again*"- **Patient**(2)
	"I want to enjoy my life, even though I've these diseases. That means, just being full of life." - **Patient***(12)*
**Social participation and independent functional capacity**	"*Sometimes I felt miserable. Because of my physical limitations, my social life was getting smaller, my work ended, and it felt like I no longer participated in society"* - **Patient** *(9)*
	"I want to go down to the dining room to eat and socialize each day", I want to continue to babysit my grandchildren every day" - **Patient** *(5)*
**Physical and disease-specific signs or symptoms assessment and control**	*"I find that promoting my physical comfort is at the heart of what I can expect from a general practitioner"* - **Patient** *(8)*

**outcome**	
**Psychological and emotional assessment and outcomes**	*What I see is that there's no psychological aid. When you are told you are a chronic patient and you have to take a drug all your life, and that this is for all your life, some people are depressed, needs psychological aid…"* - **Patient** *(12)*
**Assessment family, friends, caregivers' own health outcomes**	"*Because of her, I change everything from before. Such as my eating, my working hours, my life, my living habits, including my eating"* - **Carer***(14)*

Table 7. Discussion of review findings with community partners

Topic Discussion	Description
**Quality domains into the 3 specific categories**	• The partners see the value in this categorization, as care is complex and involves different processes and ways to measure it. One partner urges us to keep an "open mind" since we might identify an indicator that would not fall into these pre-established categories.<br>• Some partners felt there was an overlap between domains of quality and there is some repetition across the categories and we have agreed this will be refined during the formal development of indicators on the next steps of this project.
**The overlap of some quality domains between clinical processes and care outcomes**	• Care outcome is the outcome that matters to a particular patient and that might be difficult to be broadly generalized in MLTC.<br>• Reflections on how sometimes regardless of interventions provided some outcomes are probably unachievable and therefore can lead to financial and human resource waste. An example was given on how someone with severe clinical depression might not benefit from an invasive procedure such as back surgery.<br>• Partners consequently discussed how it is important to assess mental health before certain procedures, ensuring it aligns with patients outcomes of care. This has informed our discussion section on how the process of assessment and the outcome measures should be different and distinct entities.
**Screening for depression versus mental health assessment**	• Initially we had identified screening for depression as a quality domain identified in the studies. Partners felt this was too specific for those with MLTC, due to several stages and types of depression and may be less relevant to some patients.<br>• Mental health as an assessment and outcome might allow for more variability between patient's needs and priorities with care.
**The influence of health system context in defining high quality practice**	• In the United Kingdom the management of care is ideally done as a partnership between clinicians and patients, but in other European countries this might not be standard procedure. In this review, multi-centered studies have been included, and so not all 44 quality domains might be relevant to UK population. Partners question if our work will be generalizable and we acknowledged that at this stage we seek to capture specific quality of care for UK-based population.
**Identification of patients**	• Partners emphasised the importance of being formally identified as living with MLTC, as this recognition enables

**with MLTC could not be validated within thematic analysis**	healthcare professionals and specialists to anticipate the complexity of an individual's clinical presentation. For example, they highlighted the challenges posed by overlapping or conflicting symptoms across conditions, and the heightened risk of adverse effects or interactions associated with certain medications. Being identified as a person with MLTC can make it clearer for providers to have discussions about potential risks and benefits of treatment options, without being unfairly labelled as "refusing treatment," but rather understood within the broader context of personalised, risk-informed decision-making.
**Information sharing between specialists and organisations**	• This identification in patient records of MLTC prompted insights on the perceived importance information sharing between different care providers.<br>• Partners emphasised having a central point of coordination for care requirements, such as the General Practitioner within a primary care setting who serves as a gatekeeper to hold and manage comprehensive patient information. But it was also highlighted that information, such as on long-term conditions, should be available to each care provider.
**Personalised indicators in practice**	• Personalisation could mean giving people the choice to select the indicator that is most meaningful to their care or selecting the outcome of that specific indicator. Partners agreed that outcomes are generally related to enjoying life, being able to live comfortably in usual natural environment, but that could mean different things between individuals – for example the ability to work may be a top priority to some, whereas the ability to be actively mobile or engage in social activities may be more important to others.<br>• This was reflected in discussion of the importance of healthcare staff taking time to explore the unique needs of each person.
**Disease-specific indicators versus MLTC-specific indicators**	• Disease specific indicators must also be captured as relevant to each contributory condition an individual has. Quality in a person with MLTC is not an aggregate of these condition specific measures but more than that.<br>• MLTC is not a series of illnesses but needs to be acknowledged as a larger illness where its management is influenced by various processes and outcomes unique to a specific person.

References for this Supplementary file

1. Simpson G, Stuart B, Hijryana M, Akyea RK, Stokes J, Gibson J, et al. Eliciting and prioritising determinants of improved care in multimorbidity: A modified online Delphi study. J Multimorb Comorb. 2023;13:26335565231194552.
2. Giusti A, Pukrittayakamee P, Alarja G, Farrant L, Hunter J, Mzimkulu O, et al. Developing a global practice-based framework of person-centred care from primary data: a cross-national qualitative study with patients, caregivers and healthcare professionals. BMJ Glob Health. 2022;7(7).
3. Schulze J, Glassen K, Pohontsch NJ, Blozik E, Eißing T, Breckner A, et al. Measuring the Quality of Care for Older Adults With Multimorbidity: Results of the MULTIqual Project. Gerontologist. 2022;62(8):1135–46.
4. Pohontsch NJ, Schulze J, Hoeflich C, Glassen K, Breckner A, Szecsenyi J, et al. Quality of care for people with multimorbidity: a focus group study with patients and their relatives. BMJ Open. 2021;11(6):e047025.
5. Tinetti ME, Costello DM, Naik AD, Davenport C, Hernandez-Bigos K, Van Liew JR, et al. Outcome Goals and Health Care Preferences of Older Adults With Multiple Chronic Conditions. JAMA Netw Open. 2021;4(3):e211271.
6. Rijken M, Stüssgen R, Leemrijse C, Bogerd MJL, Korevaar JC. Priorities and preferences for care of people with multiple chronic conditions. Health Expect. 2021;24(4):1300–11.
7. Sasseville M, Chouinard M-C, Fortin M. Understanding patient outcomes to develop a multimorbidity adapted patient-reported outcomes measure: a qualitative description of patient and provider perspectives. Health and Quality of Life Outcomes. 2021;19(1):43.
8. Kuipers SJ, Nieboer AP, Cramm JM. Views of patients with multi-morbidity on what is important for patient-centered care in the primary care setting. BMC Family Practice. 2020;21(1):71.
9. Rimmelzwaan LM, Bogerd MJL, Schumacher BMA, Slottje P, Van Hout HPJ, Reinders ME. Multimorbidity in General Practice: Unmet Care Needs From a Patient Perspective. Front Med (Lausanne). 2020;7:530085.
10. Pereira RB, Brown TL, Guida A, Hyett N, Nolan M, Oppedisano L, et al. Consumer experiences of care coordination for people living with chronic conditions and other complex needs: an inclusive and co-produced research study. Aust Health Rev. 2021;45(4):472–84.
11. Rutten-van Mölken M, Karimi M, Leijten F, Hoedemakers M, Looman W, Islam K, et al. Comparing patients' and other stakeholders' preferences for outcomes of integrated care for multimorbidity: a discrete choice experiment in eight European countries. BMJ Open. 2020;10(10):e037547.
12. Leijten FRM, Hoedemakers M, Struckmann V, Kraus M, Cheraghi-Sohi S, Zemplényi A, et al. Defining good health and care from the perspective of persons with multimorbidity: results from a qualitative study of focus groups in eight European countries. BMJ Open. 2018;8(8):e021072.
13. Heslop L, Cranwell K, Burton T. Care coordination for chronic and complex health conditions: An experienced based co-design study engaging consumer and clinician groups for service improvement. PLoS One. 2019;14(10):e0224380.
14. Kuluski K, Peckham A, Gill A, Gagnon D, Wong-Cornall C, McKillop A, et al. What is Important to Older People with Multimorbidity and Their Caregivers? Identifying Attributes of Person Centered Care from the User Perspective. Int J Integr Care. 2019;19(3):4.
15. Manga N, Harding R, De Sa A, Murie K, Namane MK, Raubenheimer PJ, et al. Development and validation of a tool to measure patient experience in chronic disease care. Afr J Prim Health Care Fam Med. 2018;10(1):e1–e7.
16. Ferris R, Blaum C, Kiwak E, Austin J, Esterson J, Harkless G, et al. Perspectives of Patients, Clinicians, and Health System Leaders on Changes Needed to Improve the Health Care and Outcomes of Older Adults With Multiple Chronic Conditions. J Aging Health. 2018;30(5):778–99.

17. Bennett WL, Robbins CW, Bayliss EA, Wilson R, Tabano H, Mularski RA, et al. Engaging Stakeholders to Inform Clinical Practice Guidelines That Address Multiple Chronic Conditions. J Gen Intern Med. 2017;32(8):883–90.

18. Fradgley EA, Paul CL, Bryant J, Oldmeadow C. Getting right to the point: identifying Australian outpatients' priorities and preferences for patient-centred quality improvement in chronic disease care. Int J Qual Health Care. 2016;28(4):470–7.

19. Schiøtz ML, Høst D, Frølich A. Involving Patients with Multimorbidity in Service Planning: Perspectives on Continuity and Care Coordination. Journal of Comorbidity. 2016;6(2):95–102.

20. van de Pol MH, Fluit CR, Lagro J, Niessen D, Rikkert MG, Lagro-Janssen AL. Quality care provision for older people: an interview study with patients and primary healthcare professionals. Br J Gen Pract. 2015;65(637):e500–7.

21. Sav A, McMillan SS, Kelly F, King MA, Whitty JA, Kendall E, et al. The ideal healthcare: priorities of people with chronic conditions and their carers. BMC Health Services Research. 2015;15(1):551.

22. Singer SJ, Friedberg MW, Kiang MV, Dunn T, Kuhn DM. Development and preliminary validation of the Patient Perceptions of Integrated Care survey. Med Care Res Rev. 2013;70(2):143–64.

23. Bayliss EA, Edwards AE, Steiner JF, Main DS. Processes of care desired by elderly patients with multimorbidities. Fam Pract. 2008;25(4):287–93.

24. Bayliss EA, McQuillan DB, Ellis JL, Maciejewski ML, Zeng C, Barton MB, et al. Using Electronic Health Record Data to Measure Care Quality for Individuals with Multiple Chronic Medical Conditions. J Am Geriatr Soc. 2016;64(9):1839–44.

25. Tušek-Bunc K, Rakovič MP, Gorenjec NR, Klemenc-Ketiš Z. Patient perspectives on the quality of primary care for chronic conditions in Slovenia: evidence from the PaRIS survey. International Journal for Quality in Health Care. 2025;37(3):mzaf088.

26. Rijken M, van der Heide I. Identifying subgroups of persons with multimorbidity based on their needs for care and support. BMC Family Practice. 2019;20(1):179.

27. Carman KL, Dardess P, Maurer M, Sofaer S, Adams K, Bechtel C, et al. Patient and family engagement: a framework for understanding the elements and developing interventions and policies. Health Aff (Millwood). 2013;32(2):223–31.
